# Sporadic Neurofibroma of the Peritoneal Cavity

**DOI:** 10.14309/crj.0000000000000915

**Published:** 2022-11-22

**Authors:** Rahil H. Shah, Justin Forde, Kshitij Satnamdas Arora, Sean Bhalla, Sunil Amin, Shria Kumar

**Affiliations:** 1 Department of Medicine, Jackson Memorial Hospital/University of Miami, Miami, FL; 2 Division of Digestive Health and Liver Diseases at the Miller School of Medicine, University of Miami, Miami, FL; 3 Department of Pathology, Jackson Memorial Hospital/University of Miami, Miami, FL

## Abstract

Neurofibromas are peripheral nerve sheath tumors that are typically seen in syndromic conditions such as neurofibromatosis 1. We present the case of a 26-year-old woman suffering from chronic abdominal pain for over 5 years. Prior workup showed a large retroperitoneal mass extending into the abdomen and encasing multiple major vessels. She underwent endoscopic ultrasound (EUS)-guided biopsy, which was histologically consistent with a solitary neurofibroma. There is no prior report of solitary neurofibroma of the abdomen diagnosed with the use of EUS-guided biopsy. This case highlights the utility of EUS-guided biopsy in the evaluation of intra-abdominal pathology.

## INTRODUCTION

Neurofibromas are peripheral nerve sheath tumors arising from a proliferation of Schwann cells, perineural cells, endoneurial fibroblasts, and connective tissue interspersed with nerve fibers.^1^ They can be found as sporadic tumors or part of a syndrome, such as neurofibromatosis type 1 (NF1), neurofibromatosis type 2 (NF2), or type 3 multiple endocrine neoplasia (MEN). These tumors can occur in any nerve in the body and predominantly affect the cutaneous nerves of the trunk, neck, and head; in rarer cases, they have been found to involve the deep organs or the peritoneal cavity. Rarely, sites such as the colon, orbit, or mouth have been reported.^2–5^ Sporadic neurofibromas affect individuals primarily between the age of 20 and 30 years, without gender predilection.^6^ Signs and symptoms may include soft-tissue mass, pain, and focal neurologic deficits from impingement on adjacent structures. Although neurofibromas are typically benign, they have been reported to undergo transformation into malignant peripheral nerve sheath tumors, usually in patients with NF1.^6^ We present the case of a young woman with chronic abdominal pain secondary to a solitary neurofibroma of the abdominal cavity, not associated with any syndromes such as NF1, NF2, or MEN.

## CASE REPORT

**Figure 1.**
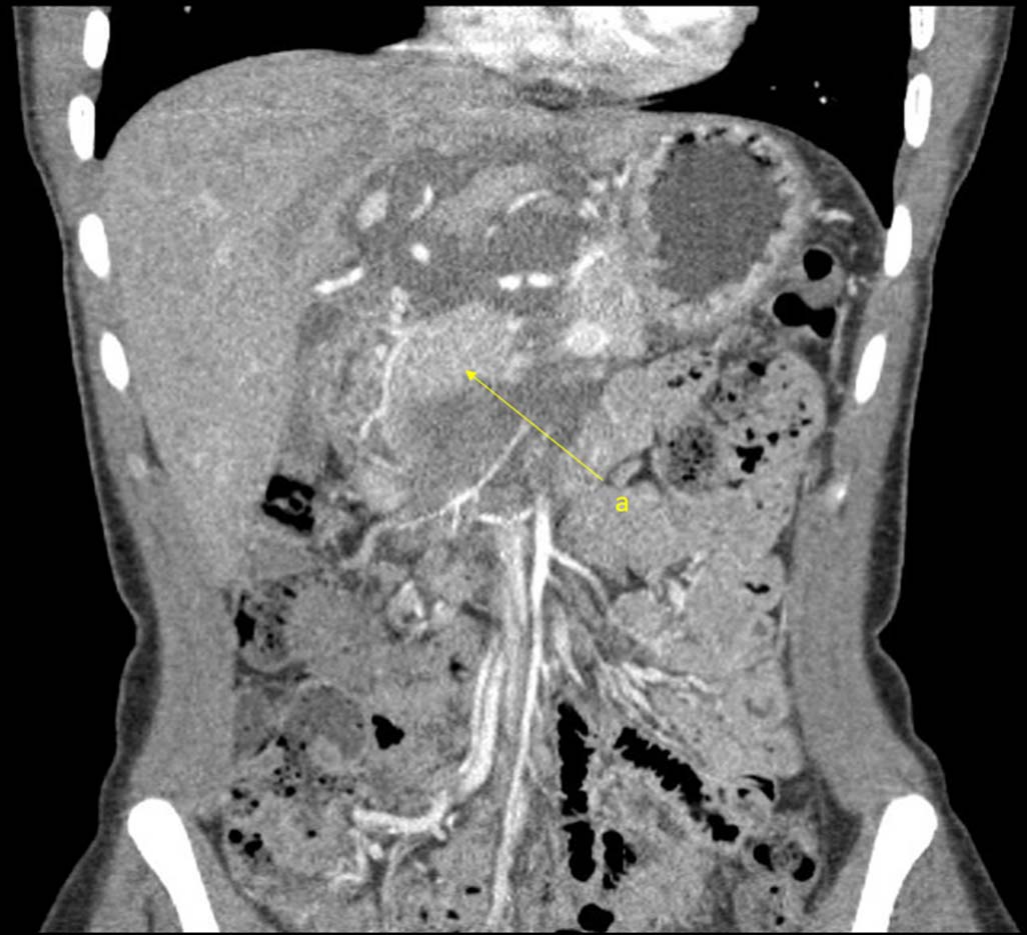
Coronal imaging using computer tomography showing a low-attenuation soft-tissue mass extending into the porta hepatis and encasing the left portal vein and pancreas (a) at the level of celiac bifurcation.

**Figure 2.**
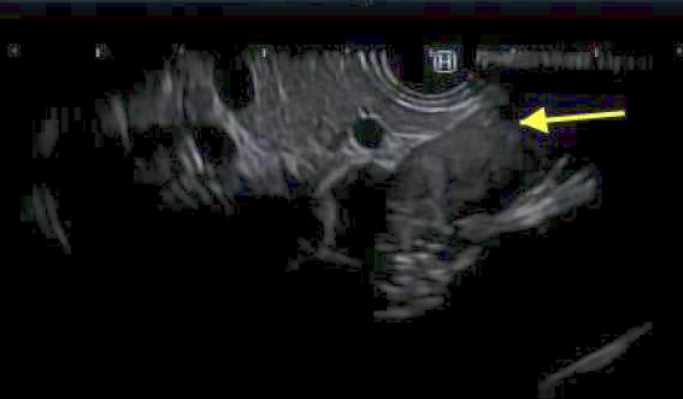
Hypoechoic lesion seen on endoscopic ultrasound images, adjacent to the pancreatic tail and aorta.

**Figure 3.**
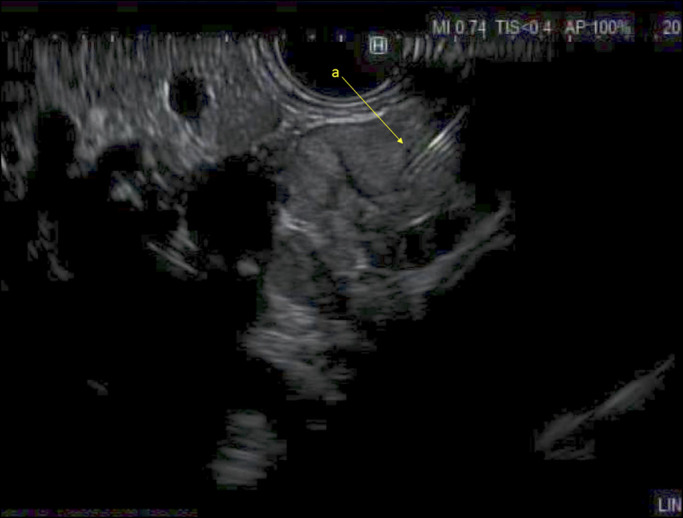
Endoscopic ultrasound-guided fine-needle biopsy of the hypoechoic lesion (a).

**Figure 4.**
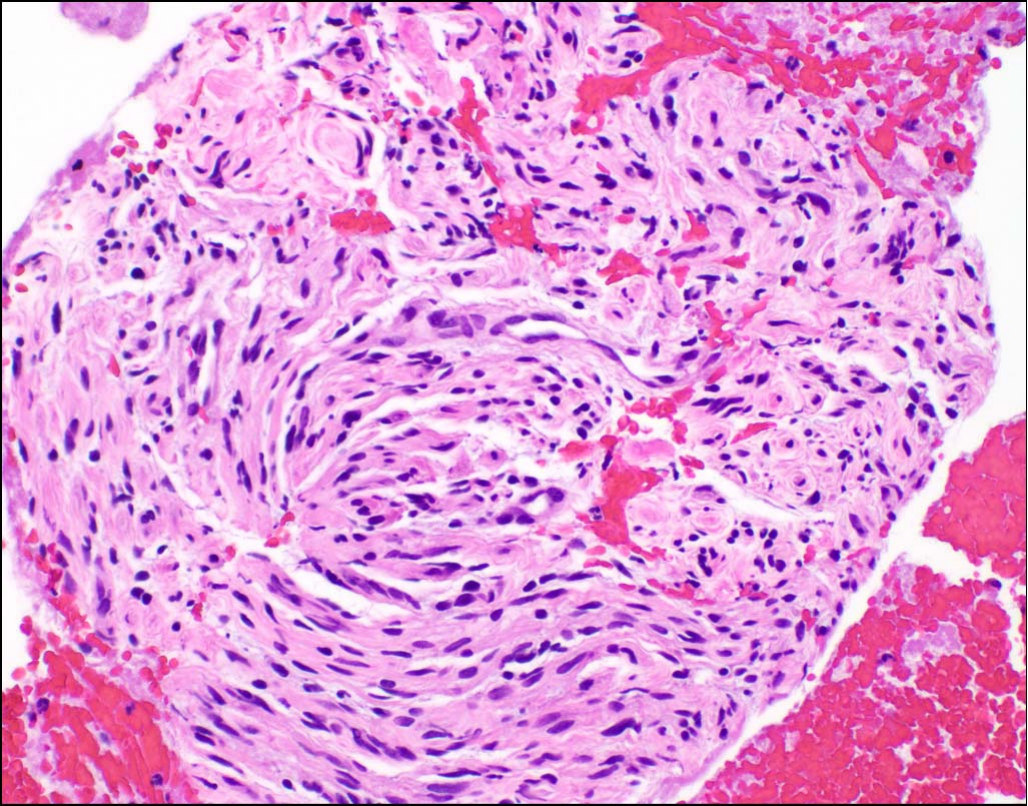
Hematoxylin and eosin staining (400×) shows scattered Schwann cells of irregular shapes, mononuclear cells, and occasional mast cells.

**Figure 5.**
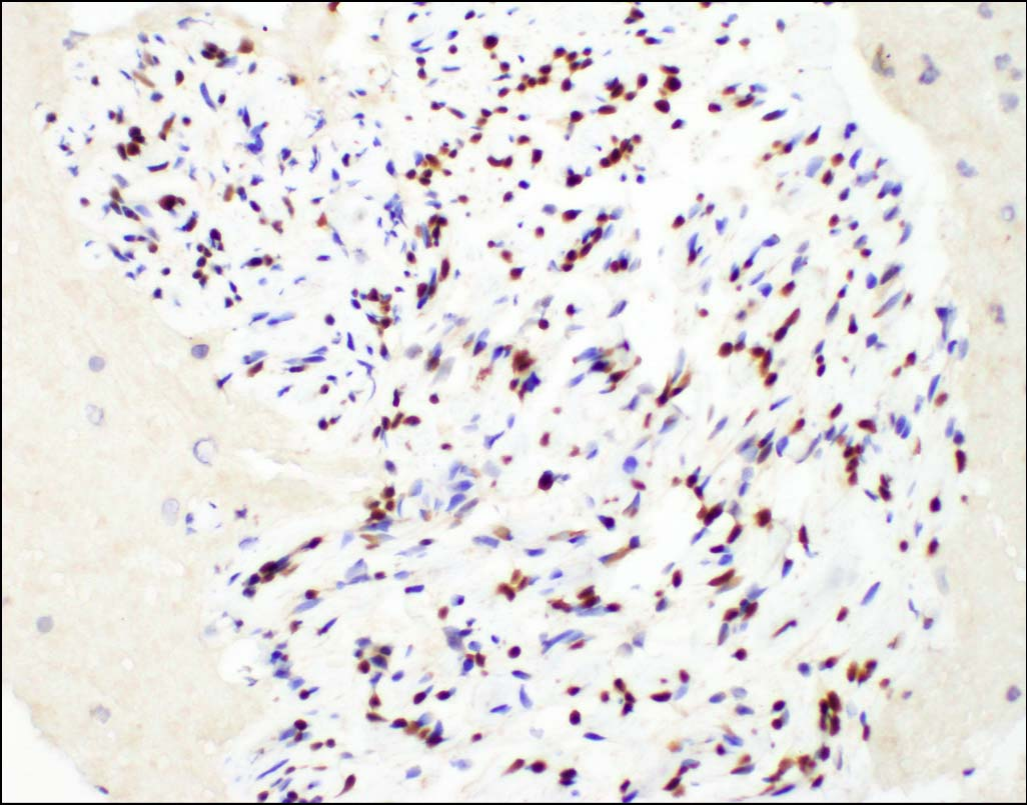
Immunohistochemical staining (400×) shows only a subpopulation of cells expressing SOX10 nuclear protein.

**Figure 6.**
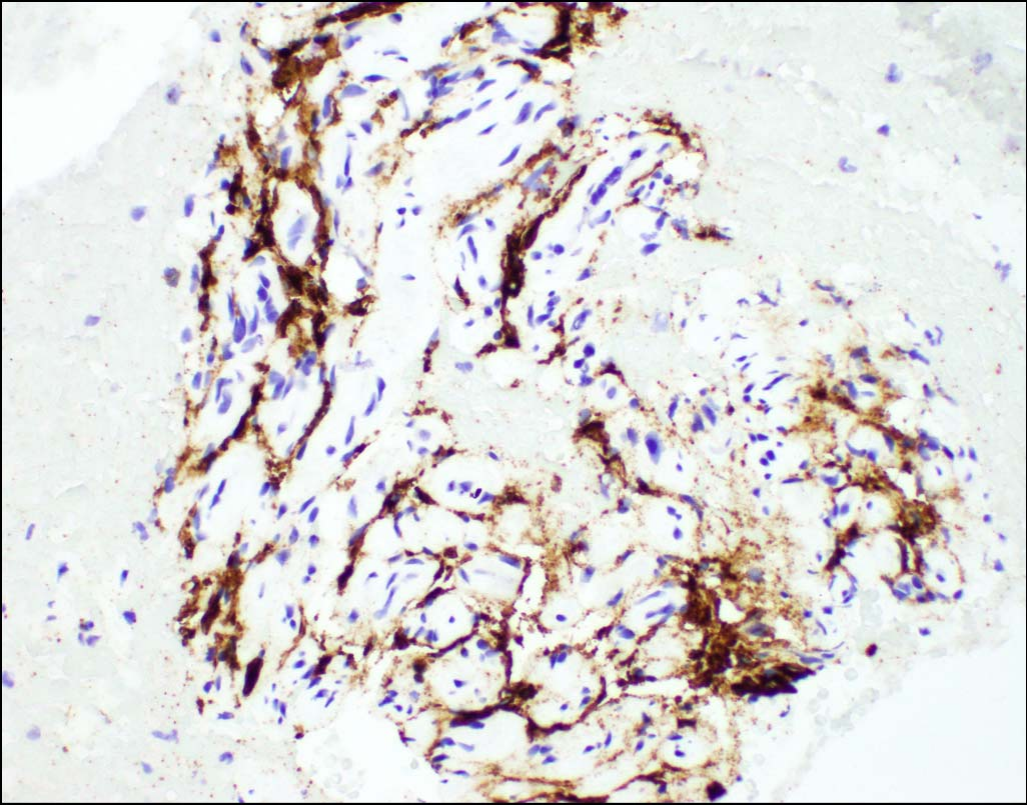
Immunohistochemical staining (400×) shows only a subpopulation of cells expressing CD34 cytoplasmic protein.

A 26-year-old woman with no significant medical history presented for chronic abdominal pain. Five years before presentation, she was found to have an abdominal mass for which she underwent both fine-needle and endoscopic ultrasound (EUS)-guided biopsies, which were nondiagnostic, but negative for malignancy. She continued to suffer from chronic abdominal pain, requiring multiple hospitalizations. During these hospitalizations, cross-sectional imaging demonstrated a 6.8 × 7.2 cm mass in the central abdomen encasing the celiac trunk, superior mesenteric artery, left portal vein and, pancreas (Figure 1). The patient underwent laparoscopy with biopsy, which was again nondiagnostic. At a subsequent follow-up, the patient complained of nausea, vomiting, and poor appetite. The mass was deemed inoperable because of its position relative to major vessels. EUS revealed a large mass measuring at least 3.7 × 1.7 cm. It appeared to be heterogeneous with hypoechoic areas (Figure 2). It was noted to be encasing the celiac and superior mesenteric arteries. The mass did not involve the pancreas. Fine-needle biopsy of the lesion was performed with 3 passes of a 22-gauge biopsy needle (Figure 3). On histology, the tumor had the morphology of neurofibroma without ganglion cells present (Figure 4). Immunohistochemistry showed that the tumor cells were focally positive for S-100, SOX10, CD34, epithelial membrane antigen, NF208, and CD 117 and negative for synaptophysin and D0G, supporting a diagnosis of neurofibroma (Figures 5 and 6). Our patient was seen by a multidisciplinary team, and the decision was made to continue to manage conservatively without any plans for mass debulking, with plans for symptomatic pain control.

## DISCUSSION

We present a rare case of a patient diagnosed with a sporadic intra-abdominal neurofibroma causing chronic abdominal pain. Our patient's presentation mirrors that of other patients with abdominal neurofibromas with insidious onset and nonspecific symptoms such as abdominal pain, cramping, or lower back pain. A prior literature review found only 7 cases of solitary neurofibromas in the mesentery, which were predominantly in the ileum, without a diagnosis of NF1.^7^

Despite multiple imaging studies and multiple biopsies, including a laparoscopic biopsy, a diagnosis was not established until a second EUS-guided biopsy was performed. Imaging is often the initial modality used for evaluating these peripheral nerve tumors and is frequently performed because of symptoms such as pain or nerve dysfunction, whether motor or sensory.^8^ Despite the useful nature of cross-sectional imaging, it is not sufficient to establish a diagnosis. EUS is a useful diagnostic procedure that can allow for increased characterization of intra-abdominal lesions and a safe noninvasive method for tissue acquisition. In a case of colonic solitary neurofibroma, EUS was used to measure the depth of invasion.^5^ To the best of our knowledge, EUS-guided biopsy has not been reported to diagnose peritoneal neurofibromas.

The lifetime risk of developing malignant peripheral nerve sheath tumors in patients with NF1 has been cited at 8%–13% making biopsy particularly important, especially when there is rapid enlargement of the soft-tissue mass, change in texture, pain, and neurological deficit concerning for malignancy.^9^ Consensus recommendations for tissue acquisition include options such as fine-needle aspiration, needle biopsy, open incisional biopsy, and excisional biopsy.^10^ Fine-needle aspiration tends to lead to loss of architectural relationships. Open incisional biopsies are highly invasive and often limited to tissue samples of only 1 site.^10^ Excisional biopsy requires removing the entire tumor which may be limited by the size or location of the tumor. The preferred modality for tissue acquisition is needle biopsy, which can be obtained using EUS.

On histology, neurofibromas are encapsulated tumors consisting of fibroblasts with serpentine, hyperchromatic nuclei that are separated by fine collagen fibers.^11^ They are diffusely positive for S-100 and SOX10, differentiating them from myxomas, and they lack calretinin and stain positive for CD34 and factor XIIIa, which differentiates them from schwannomas.^11^

The management of peripheral nerve tumors is predominantly surgical—although not all patients require surgery. Surgical indications include neurological deficit, bleeding, pain, disfiguring lesions, and confirmed or suspected malignancy.^10^ When surgery is required, as in the case of malignant peripheral nerve sheath tumors, the aim is complete removal of the lesion with tumor-free margins with preoperative or postoperative adjuvant radiation or chemotherapy.^10^ Surgical options may be limited or due to the involvement of nearby structures or entwinement of nerve fascicles. Benign neurofibromas may be managed with clinical and radiological observation, although there are no consensus guidelines for imaging modality or frequency. On long-term follow-up, tumors have been noted to usually remain stable and, in a rare occasion, spontaneously decrease.^12^ To the best of our knowledge, this is the first case report of a peritoneal solitary neurofibroma without a concurrent syndromic state such as NF1, NF2, or MEN, which was diagnosed by EUS-guided biopsy.

## DISCLOSURES

Author contributions: RH Shah, J. Forde, and KS Kumar all were involved in initial manuscript preparations, editing, and preparation for publishing. S. Bhalla and S. Amin were involved in editing and preparation for publication. S. Arora was involved in preparation of figures and captions for histopathology slides. KS Kumar is the article guarantor.

Financial disclosure: None to report.

Previous presentation: This case was presented at University of Miami, Miami, FL 8th Annual Eugene J. Sayfie, MD, Research Day; May 22, 2022.

Informed consent was obtained for this case report.
